# The early outcomes of candidates with portopulmonary hypertension after liver transplantation

**DOI:** 10.1186/s12876-018-0797-8

**Published:** 2018-06-07

**Authors:** Bingsong Huang, Yi Shi, Jun Liu, Paul M. Schroder, Suxiong Deng, Maogen Chen, Jun Li, Yi Ma, Ronghai Deng

**Affiliations:** 1 Organ Transplant Center, the First Affiliated Hospital, Sun Yat-sen University, No. 58 Zhongshan 2nd Road, Guangzhou, 510080 China; 2 Department of Respiratory, the First People’s Hospital affiliated to Guangzhou Medical University, Guangzhou, 510080 China; 3 Department of Surgery, Duke University Medical Center, 10 Duke Medicine Circle Durham, Durham, NC 27710 USA

**Keywords:** Portopulmonary hypertension, Liver transplantation, Meta-analysis

## Abstract

**Background:**

Portopulmonary hypertension (PPH) was once regarded as a contraindicaton to liver transplantation (LT). However, growing evidence has indicated that PPH patients undergoing LT may show similar outcomes compared to those without PPH, and researchers have recommended it not be an absolute contraindication. Given this controversy, we aimed to identify and review the current evidence on this topic and to provide a comparison of the outcomes after LT between candidates with PPH and those without.

**Methods:**

We systematically searched the MEDLINE, EMBASE and Cochrane Library databases for all studies that compared the outcomes of PPH patients and those without PPH after LT. All studies reporting outcomes of PPH patients versus those without PPH (Control) were further considered for inclusion in this meta-analysis. Odds ratios (OR) and 95% confidence intervals (CI) were calculated to compare the pooled data between PPH and Control groups.

**Results:**

Eleven retrospective trials and one prospective, randomized, controlled trial, involving 37,686 transplant recipients were included. The PPH patients had increased 1-year mortality with an OR of 1.59 (95% CI = 1.26–2.01, *P* = 0.0001) compared to the control group. There was no significant difference in graft loss and 30-day mortality after LT between the two groups.

**Conclusions:**

Patients with PPH who underwent LT had increased 1-year mortality compared to those without PPH, while graft loss and 30-day mortality were similar. Nevertheless, LT may be a reasonable therapeutic option for some patients with PPH, but further studies are needed to identify those select patients with PPH who would benefit most from LT.

**Electronic supplementary material:**

The online version of this article (10.1186/s12876-018-0797-8) contains supplementary material, which is available to authorized users.

## Background

Portopulmonary hypertension (PPH) is defined by the presence of the following features in patients with portal hypertension: mean pulmonary arterial pressure (mPAP) determined by Portopulmonary hypertension (PPH) is defined by the presence of the following features in patients with portal hypertension: mean pulmonary arterial pressure (mPAP) determined by right-heart catheterisation of > 25 mmHg at rest or > 30 mmHg during exercise, elevated pulmonary vascular resistance (PVR) > 3 wood units (240 dynes/s per cm-5), and normal pulmonary artery wedge pressure (PAWP) < 15 mmHg. [1–3]. PPH represents a serious complication of portal hypertension and is regarded as a contraindication to liver transplantation (LT) by some experts. It is a relatively common pathologic state in the setting of end-stage liver disease (ESLD), especially cirrhosis, with a reported incidence ranging from 1 to 10% to as high as 39% in patients receiving LT [4–6].

The pathophysiology of the relationship between portal hypertension and pulmonary hypertension is poorly understood. Some researchers consider the development of pulmonary hypertension in the ESLD population dependent upon the presence of significant portal hypertension, but the mechanisms by which portal hypertension cause pulmonary hypertension remain unclear [7]. Others maintain that the elevation of mPAP represents a hyperdynamic state of blood circulation and that this elevation in mPAP eventually leads to portal hypertension [4, 8–11]. The true pathophysiology likely represents a combination of factors including the patient’s underlying disease process and relevant comorbidities that determine the dominant causes of these high-pressure states.

Recently growing evidence suggests that PPH should no longer be considered an absolute contraindication to LT unless PPH is severe and associated with right ventricular dysfunction [6, 12, 18, 19]. Newer retrospective comparisons between patients with PPH and those without who underwent LT showed similar mortality between the two groups or slightly higher rates of death in PPH group that did not reach statistical significance [13, 14]. In 2012, a prospective controlled study showed that there were no significant differences between the PPH group and the control group in terms of six-month patient and graft survivals (100% vs. 88.9, 100% vs. 100%, respectively) [15].

The role of LT in patients with PPH has long been debated. Given the current controversy, in this meta-analysis, we aimed to combine data from all published studies to reevaluate the outcomes of the LT patients with preoperative PPH and give some suggestions for the management of PPH patients waiting for LT.

## Methods

### Data sources and searches

We searched the PubMed/Medline, Embase and Cochrane library databases using the terms “Portopulmonary hypertension” OR “Pulmonary hypertension” AND “Liver transplantation.” The search included all studies published up to February 2017. Publications were limited to those reporting results from human subjects. Review articles were excluded after limit filtering. To prevent missing relevant publications on the topic, we also performed manual searches of the references of the relevant publications.

### Study selection: Inclusion and exclusion criteria

Only those reporting outcomes of LT patients preoperatively diagnosed with PPH were included. Overlapping cohort studies from the same institution were excluded to avoid duplication. Studies lacking a control group or whose populations included subjects with other pulmonary diseases were excluded since other pulmonary disease processes can cause pulmonary hypertension. Studies comparing outcomes between the PPH patients with LT and without LT were also excluded. Studies for which the data could not be extracted for analysis such as those reporting outcomes only in figure format (without description or tables) were excluded. Case reports whose data had poor homogeneity and studies examining patients who received multi-organ transplants were also excluded.

### Quality assessment and data extraction

Publications were reviewed and two independent investigators extracted data with disagreements being resolved through discussion and consensus. The primary outcome was overall one-year patient survival rates. Secondary outcomes included early (30 days post-LT) patient and graft survival rates. The Newcastle-Ottawa quality assessment scale (Additional file 1: Table S1) [16] was applied to assess the quality of all included trials. A study can be awarded a maximum of one star for each numbered item within the Selection and Outcome categories. A maximum of two stars can be given for Comparability. Articles scoring five stars or more were considered to be of high quality. In addition, a RCT trial included was assessed by the Jadad score [17] in which case a score of at least 4 indicated a high methodological quality.

### Data synthesis and analysis

Pooled odds ratios (OR) were used to evaluate the event rates, and the results were reported with 95% confidence intervals (CI). A *P* value < 0.05 was considered a significant difference in the values between the two groups. Heterogeneity through all the included studies was evaluated by χ^2^ and *I*^*2*^ statistical tests. Heterogeneity was considered significant when *P* < 0.05 or *I*^*2*^ > 50%, and a random effect model was adopted. A random effect model is a kind of hierarchical linear model, which assumes that the data set being analyzed consists of a hierarchy of different populations whose differences relate to that hierarchy. When *P* > 0.05 for χ^2^ or *I*^*2*^ < 50% for *I*^*2*^ statistical tests, indicating low statistical heterogeneity in both cases, a fixed effect model was used. A fixed effect model is a statistical model that represents the observed quantities in terms of explanatory variables that are treated as if the quantities were non-random. A funnel plot was used to assess publication bias. A funnel plot is designed to check the existence of publication bias in systematic reviews and meta-analyses. The largest studies will be near the average while small studies will be spread on both sides of the average. Variation can indicate publication bias. All statistical analyses for the current study were performed with Review Manager (RevMan Version 5.3.5, The Nordic Cochrane Centre, The Cochrane Collaboration, 2014).

## Results

### Search results and included studies

**Fig. 1:**
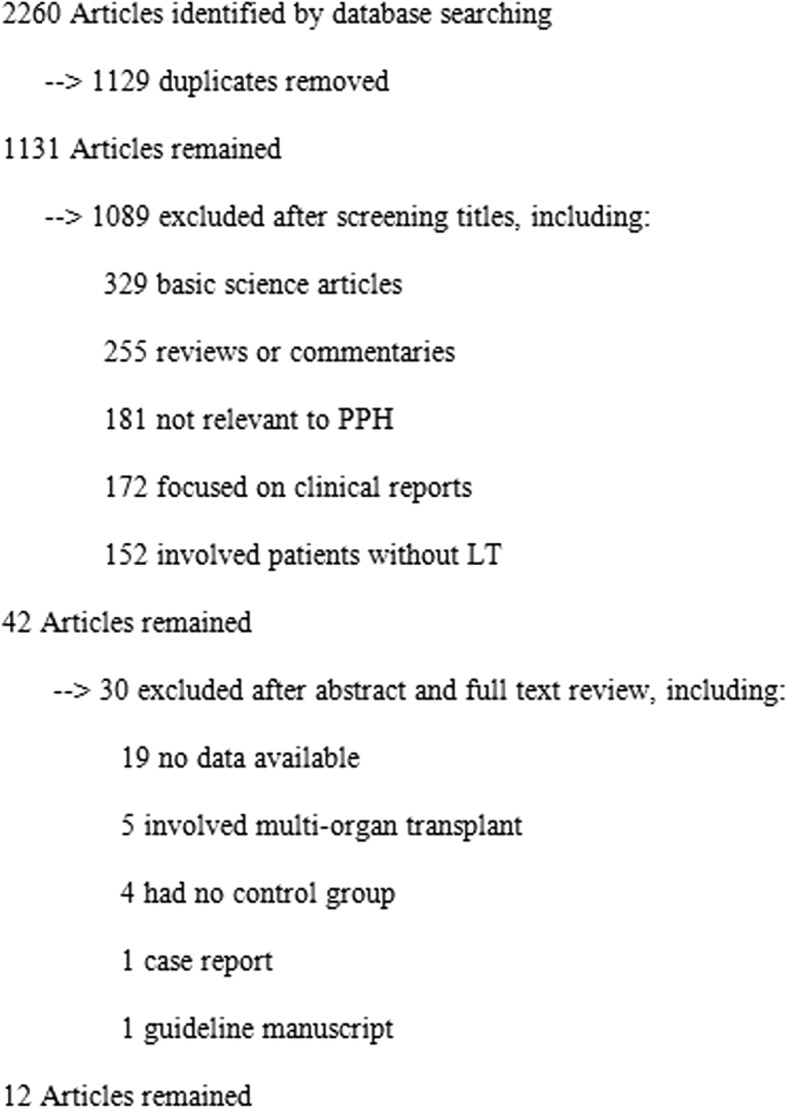
PRISMA flow diagram showing selection of articles for review

The PRISMA flow diagram and results based on the search strategies and selection criteria described above are outlined in Fig. 1. Briefly, 2260 articles were initially identified. Among those references, 2218 studies were excluded after screening titles. The remaining 42 publications reporting results after LT for patients with PPH underwent more extensive review. Nineteen of these studies had no data available and were excluded from this meta-analysis. Five studies involved multiple organ transplantation, 4 studies lacked a control (no-PPH) group, one study was a case report, and one study was a manuscript reporting guidelines, which were also excluded. A total of 12 studies meeting all criteria were included in this meta-analysis, and the study characteristics are shown in Table 1. No evidence of publications bias among the included studies was found by means of a funnel plot (data not shown). A total of 507 LT recipients with PPH and 37,179 LT patients without PPH were included in this meta-analysis.

**Table 1:** Characteristics of the PPH trials

References	Institute	Sample size		Study periods	Recipients age	MELD score		NOS star level
		PPH	No-PPH			PPH	No-PPH	
DeMartino(2017) [4] Rajaram(2016) [13]	USA(single center) USA(single center)	31 13	269 20	2010–2013 2005–2015	57 (50–62) 52(37–62)	32 (25–38) 21.0 ± 9.2	25 (20–29) 24.7 ± 9.5	66
Bozbac(2015) [17] Salgia (2014) [6] Mangus(2013) [16]	Turkey(single center) SRTR^a^ USA(single center)	47 78 102	156 34,240 1161	2004–2015 2002–2010 2001–2010	42.1 ± 14.1 54 (49–60) 53 (18–76)	N/A^b^ 14 (11–18) 22(9–40)	N/A 18 (13–25) 18 (6–40)	6 6 7
Yassen(2012) [15] ▲ Pietri(2010) [14] Saner (2006) [7]	Egypt(single center) Italy(single center) Germany(single center)	9 24 23	10 24 48	2008–2011 2003–2008 2004–2005	50.3 54(49–60) 49.6	17 ± 5 25.0 ± 12.0 N/A	14 ± 2 22.0 ± 10.9 N/A	◆5 6 6
Veloso(2004) [21] Starkel (2002) [18] Ramsay(1997) [32] Taura(1996) [19]	Brazil(single center) UK(single center) USA(single center) Spain(single center)	31 38 103 8	26 107 1103 15	1999–2001 1997–1999 1984–1995 N/A	46 49.2 N/A 45.2	N/A N/A N/A N/A	N/A N/A N/A N/A	6 6 5 5

▲, random controlled, double-blind study**; ◆**Jadad **score**

^a^SRTR, Scientific Registry of Transplant recipients

^b^N/A, non-available

### Hemodynamic parameters in the PPH group

The diagnosis of PPH is made from measurements during right heart catheterization with mPAP of > 25 mmHg, PVR > 240 dynes∙s∙cm − 5, and PAWP < 15 mmHg, and this definition was relatively consistent among the trials included in this meta-analysis. Some of the articles used a higher threshold of mPAP for diagnosis (mPAP> 30 mmHg) and inclusion in the PPH group [18, 19]. Others such as the DeMartino 2017 article, only included patients with moderate to severe PPH (mPAP> 35 mmHg and PVR greater than 240 PVR dynes∙s∙cm − 5) [4]While many studies used a single value of mPAP to serve as inclusion criteria for their PPH group, some further separated the PPH group into three grades of PPH: mild, moderate, and severe with considerable variation in the distinction between the three subgroups among the trials included in this meta-analysis. The hemodynamic parameters of the PPH groups in each of the studies are shown in Table 2.

**Table 2:** Hemodynamics condition of PPH group

References	Grade of PPH(mmHg),n			Mean mPAP(mmHg)
DeMartino(2017) [4]	> 35			38(range,35–46)
	31			
Rajaram(2016) [13]	> 25			45.51 ± 2.1
	13			
Bozbac(2015) [17]	> 30			44.2 ± 7.8
	47			
Salgia (2014) [6]	25–35(treated PPH)			N/A
	78			
Mangus(2013) [16]	25–30(low mild)	30–34(high mild)	> 35(moderate)	N/A
	63	30	9	
Yassen(2012) [15]	25–34(mild)	35–44(moderate)		30.1 ± 11.4
	6	3		
Pietri(2010) [14]	25–34(mild)	> 35(moderate)		N/A
	21	3		
Saner (2006) [7]	25–34(mild)	35–44(moderate)	> 45(severe)	N/A
	16	5	2	
Veloso(2004) [21]	> 25			31.48 ± 4.42
	31			
Starkel (2002) [18]	25–34(mild)	> 35(moderate to severe)		N/A
	31	7		
Ramsay(1997) [32]	30–44(mild)	45–59(moderate)	> 60(severe)	N/A
	81	14	7	
Taura(1996) [19]	> 25			33.4(range-28-38)
	8			

### Primary outcome

#### 1-year mortality

**Fig. 2:**
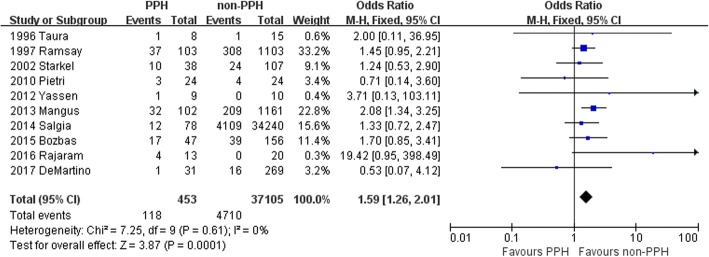
Patient mortality at 1 year

Ten studies involving 453 LT recipients with PPH and 37,105 LT recipients without PPH reported 1-year survival rates after transplantation. The results of the SRTR study (2014 Salgia, et al.) showed a higher 1-year mortality rate in PPH patients compared to patients without PPH (*P* = 0.005) [6]. In the remaining 9 studies [5, 13, 15–19], there was no significant difference in 1-year mortality rates between the two groups. The combined 1-year mortality rate was 26.0% for the PPH group and 12.7% for the control group. There was no significant heterogeneity identified among the 10 studies (χ^2^ = 7.25, *p* = 0.61, *I*^2^ = 0%). A fixed effect model was used, which showed that the OR for mortality at 1 year after LT was 1.59 (95% CI =1.26–2.01; *P* = 0.0001) for the PPH group compared to the control group (Fig. 2). Thus, one-year mortality after LT was significantly higher in the PPH group compared to the control group.

### Secondary outcomes

#### 30-day mortality

**Fig. 3:**
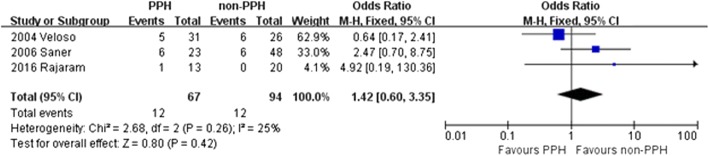
Patient mortality at 30 days

There were 3 studies that reported 30-day mortality after LT. Overall, the mortality within 30 days of LT surgery was 12.8% for the control group and 17.9% for the PPH group. No heterogeneity was identified across the 3 studies (χ^2^ = 2.68, *P* = 0.26; *I*^2^ = 25%), thus a fixed effect model was adopted. The OR for 30-day mortality in liver recipients with PPH versus those without was 1.42 (95% CI = 0.60–3.35, *P* = 0.42), which was not statistically significant (Fig. 3). These data show no significant difference in 30-day mortality after LT between those with PPH and those without.

#### Graft loss rates

**Fig. 4:**
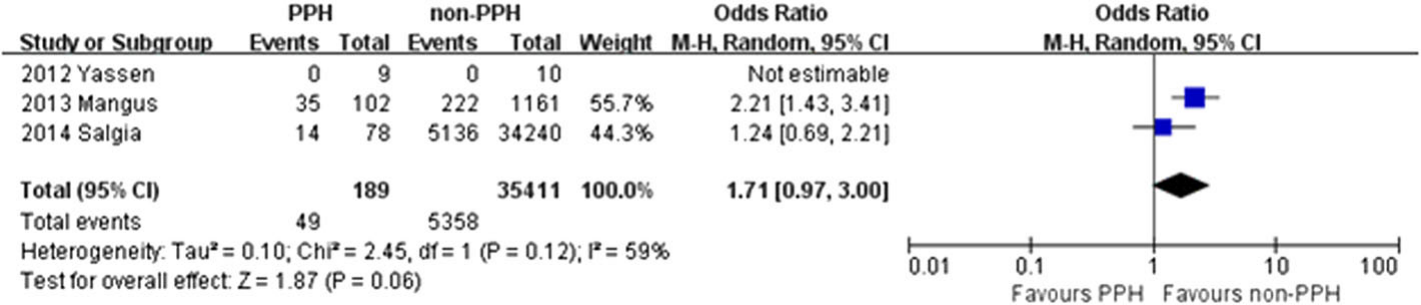
Graft loss at one year

Another secondary endpoint that was examined was graft loss at 1 year after transplantation in order to understand the influence of initial PPH on the success of the transplant. Three of the studies reported graft loss rate. There was no heterogeneity detected among the three studies that reported one year graft loss rates (χ^2^ = 2.45, *P* = 0.12; *I*^2^ = 59%), thus a random effect model was implemented for further comparison. The OR for one-year graft loss was 1.71 (95% CI =0.97–3.00, *P* = 0.06) in the patients with PPH compared to those without (Fig. 4). Therefore, patients without PPH demonstrated significantly better one-year graft survival after LT than those that had PPH prior to transplant.

## Discussion

In this manuscript we aimed to provide a comprehensive meta-analysis comparing the early outcomes after LT between candidates with PPH and those without. A total of twelve studies with 37,688 patients were included in this meta-analysis. The primary outcome of 1-year mortality was significantly higher in those with PPH, while 30-day mortality was no different from controls. In addition, those with PPH had significantly higher rates of graft loss at 1 year after LT. To our knowledge this is the first comprehensive, systematic review of the available data on this topic and therefore provides important insight into the controversy regarding the benefit of LT in patients with PPH.

Patients with liver disease are at higher risk for pulmonary vascular conditions such as PPH due to systemically high intravascular flow and increased pulmonary venous volume. In a communication from the French pulmonary arterial hypertension (PAH) registry (*n* = 154, where only 33% had been treated with PAH-specific therapies), Le Pavec, et al. [20] described 1-, 3-, and 5-year survivals of 88, 75 and 68%, respectively, for PPH patients. Causes of death in this study were equally distributed between right ventricle failure due to progressive PPH and direct complications from liver cirrhosis. If no therapy is implemented, the prognosis of PPH is very poor with 5-year survival of 4–14% reported at some centers [18, 21]. Current pharmacologic treatment options for PPH include oral and intravenous vasodilator therapy such as prostacyclin analogues, phosphodiesterase 5-inhibitors, and endothelin receptor antagonists [1]. Nevertheless, the available data supporting the use of these specific therapies in PPH are only presented in case series and uncontrolled observational studies.

The therapeutic potential of LT in PPH has also been demonstrated, as Bozbas, et al. showed significant reductions in mPAPs after LT in patients with PPH [17]. However, due to poor outcomes reported early on in the experience of LT for patients with PPH, it was considered a contraindication to LT by many transplant centers [22–25]. More recent evidence from retrospective data in multiple centers suggests that unless PPH is severe and associated with right ventricular dysfunction, it should no longer be considered an absolute contraindication to liver transplant [4, 6, 17, 26]. Indeed, Ramsay, et al. reviewed 1205 LT recipients involving 102 PPH patients, the 3-year mortality rates of the no-PPH, mild PPH, moderate PPH and severe PPH groups were 28, 33, 35, and 71%, respectively [18]. They concluded that patients with severe PPH likely had pathological changes in the pulmonary vasculature that were irreversible even after LT, as severe PPH was associated with a much higher perioperative mortality rate. A retrospective study from the UNOS SRTR database reported 123 PPH patients [6], seventy-eight of them underwent LT, whose 1- and 3-year survival were 85 and 81%. The other 45 had not been transplanted and 11 of them died on the waitlist, which indicated 3-year survival for PPH patients on the waitlist was about 75.6%. Based in part on these data, the United States organ allocation policy gives higher priority to perform LT in PPH patients if hemodynamics are expected to significantly improved and meet standardized Model for end-stage liver disease (MELD) exception guidelines [4, 27, 28]. Because of the important prognostic implications demonstrated by these studies along with these changes in organ allocation policies, the American Association for the Study of Liver Disease and the International Liver Transplantation Society recommended all patients evaluated for LT be screened for PPH by transthoracic echocardiogram (TTE), with confirmatory testing by right heart catheterization (RHC) [3, 8, 29, 30].

The overall mortality at 1 year after LT in our meta-analysis is comparable to the individual analyses with 26% mortality in the PPH group. However, this data includes multiple degrees of PPH as demonstrated in our analysis of the hemodynamic status of the patients included in each of the studies. The individual studies such as the Ramsay, et al. and Saner, et al. that defined three groups mild, moderate, and severe PPH prior to LT can help to determine and select the appropriate patient population that would have outcomes similar to those without PPH. Identifying threshold values of mPAP in PPH patients that predict poorer outcomes after transplant or provide guidance about which patients would benefit from specific treatment of their PPH prior to LT will be useful questions for future work in this field. Determination of these values and novel ways of evaluating prognosis after LT in the population of patients with PPH also has broader implications for organ allocation policy in this patient population.

There are multiple limitations inherent to this meta-analysis. The majority of the studies that were included for analysis are retrospective, observational studies. In addition, all but four of the studies had relatively small sample sizes of participants, which may have precluded an accurate assessment of heterogeneity. Donor factors such as preoperative condition of the donor, age, cold and warm ischemia time, and donation type have a great impact on the post-operative outcomes of LT recipients. For instance, donation after cardiac death (DCD) LT has worse long-term outcomes compared to donation after brain death (DBD) LT, with an increase in biliary complications, ischemic cholangiopathy, graft loss and mortality [31]. However, most of studies included in this meta-analysis did not provide donor information (donor baseline was only described in the studies of Salgia, et al. and Mangus, et al.), which may represent a confounding variable that could influence the comparison of mortality rates between groups. Prior to the implementation of TTE as a valuable tool in identifying patients with PPH, this technology was not routinely used to screen LT candidates for these phenomena. Therefore, a portion of candidates who suffered from PPH may be missing from our analysis, especially in those studies that reported data from earlier times. In addition, the follow-up time in our analysis was relatively short (only out to 1 year), which may not be representative of the long-term outcomes for these patients after LT. Also, there were only 3 studies (with relatively small sample sizes) reporting 30-day mortality so drawing conclusions based on this analysis is difficult. Due to the wide distribution of study periods and the advances in technology and operative techniques over these time periods, a degree of bias related to these temporal changes in management may have been present and was not accounted for in the analysis. Lastly, a few of the studies had no available baseline data such as MELD score and age of patients included calling into question the quality of those studies. However, all the studies were evaluated by the Newcastle-Ottawa quality assessment scale or similar evaluation method and determined to be of sufficient quality for this meta-analysis. 

## Conclusion

There is an increase in 1-year patient mortality and graft loss after LT in candidates with PPH. Thus, PPH remains an important risk factor that should continue to be screened for in LT candidates. It is likely that different grades of PPH exist some of which are amenable to treatment, and select patients with PPH are likely to benefit more than others from LT. Therefore, more randomized controlled trials with a larger sample sizes and long term follow-up are needed to evaluate the long-term outcomes in these patients as well as to refine the selection of patients with PPH who would benefit most from LT.

## Additional file

Additional file 1 : **Table S1**. Newcastle - Ottwa Quality Assessment Scale. (DOCX 14 kb)

## Abbreviations

CI: Confidence intervals
DBD: Donation after brain death
DCD: Donation after cardiac death
ESLD: End-stage liver disease
HR: Hazard ratio
LT: Liver transplantation
MELD: Model for end-stage liver disease
OR: Pooled odds ratios
PAH: Pulmonary arterial hypertension
PAP: Pulmonary arterial pressure
PPH: Portopulmonary hypertension
RHC: Right heart catheterization
SRTR: Scientific registry of transplant recipients
TTE: Transthoracic echocardiogram
