# Supplementary material for: The early outcomes of candidates with portopulmonary hypertension after liver transplantation
Source: BMC Gastroenterol. 2018 Jun 7;18:79. doi: 10.1186/s12876-018-0797-8 (PMC5992875; doi:10.1186/s12876-018-0797-8)
Supplement: Supplementary file 1 — : Table S1. Newcastle - Ottwa Quality Assessment Scale. (DOCX 14 kb) [file 12876_2018_797_MOESM1_ESM.docx]

Table S1. Newcastle - Ottwa Quality Assessment Scale

Note: A study can be awarded a maximum of one star for each numbered item within the Selection and

Exposure categories. A maximum of two stars can be given for Comparability.

Selection

1) Is the case definition adequate?

a) yes, with independent validation ¯

b) yes, eg record linkage or based on self reports

c) no description

*Star if the PPH is defined with accurate hemodynamics conditions.

2) Representativeness of the cases

a) consecutive or obviously representative series of cases ¯

b) potential for selection biases or not stated

*Star if demographics and hemodynamics conditions are both clearly explained

3) Selection of Controls

a) community controls ¯

b) hospital controls

c) no description

4) Definition of Controls

a) no history of disease (endpoint) ¯

b) no description of source

*Star if control protocol has conducted a good match with other demographics and basic disease

Comparability

1) Comparability of cases and controls on the basis of the design or analysis

a) study controls for _______________ (Select the most important factor.) ¯

b) study controls for any additional factor ¯ (This criteria could be modified to indicate specific

control for a second important factor.)

*Star a) if PPH group has a exact rules of inclusion just as Selction 1);

*Star b)if Cox regression is used to examine potential associations between PPH and post-transplant outcomes.

Exposure

1) Ascertainment of exposure

a) secure record (eg surgical records) ¯

b) structured interview where blind to case/control status ¯

c) interview not blinded to case/control status

d) written self report or medical record only

e) no description

2) Same method of ascertainment for cases and controls

a) yes ¯

b) no

3) Non-Response rate

a) same rate for both groups ¯

b) non respondents described

c) rate different and no designation
